# Development and Evaluation of a Clinician-Vetted Dementia Caregiver Resources Website: Mixed Methods Approach

**DOI:** 10.2196/54168

**Published:** 2024-04-04

**Authors:** Jaye E McLaren, Dat Hoang-Gia, Eugenia Dorisca, Stephanie Hartz, Stuti Dang, Lauren Moo

**Affiliations:** 1 New England Geriatric Research Education and Clinical Center, Veterans Affairs Bedford Health Care System Bedford, MA United States; 2 Palo Alto Geriatric Research Education and Clinical Center, Veterans Affairs Palo Alto Health Care System Palo Alto, CA United States; 3 Bronx Geriatric Research Education and Clinical Center, Veterans Affairs Bronx Health Care System Bronx, NY United States; 4 Eastern Colorado Geriatric Research Education and Clinical Center, Eastern Colorado Veterans Affairs Health Care System Aurora, CO United States; 5 Miami Geriatric Research Education and Clinical Center, Miami Veterans Affairs Health Care System Miami, FL United States; 6 Division of Geriatrics and Palliative Care, University of Miami Miller School of Medicine Miami, FL United States; 7 Elizabeth Dole Center of Excellence for Veteran and Caregiver Research, US Department of Veterans Affairs Washington, DC United States; 8 Cognitive Behavioral Neurology, Massachusetts General Hospital, Harvard Medical School Boston, MA United States

**Keywords:** Alzheimer disease, caregiver education, dementia, interdisciplinary, older adults, virtual resources, website development

## Abstract

**Background:**

About 11 million Americans are caregivers for the 6.7 million Americans currently living with dementia. They provide over 18 billion hours of unpaid care per year, yet most have no formal dementia education or support. It is extremely difficult for clinicians to keep up with the demand for caregiver education, especially as dementia is neurodegenerative in nature, requiring different information at different stages of the disease process. In this digital age, caregivers often seek dementia information on the internet, but clinicians lack a single, reliable compendium of expert-approved digital resources to provide to dementia caregivers.

**Objective:**

Our aim was to create a dementia caregiver resources website to serve as a hub for user-friendly, high-quality, and expert-reviewed dementia educational resources that clinicians can easily supply to family caregivers of people with dementia.

**Methods:**

An interdisciplinary website development team (representing dementia experts from occupational therapy, nursing, social work, geriatrics, and neurology) went through 6 iterative steps of website development to ensure resource selection quality and eligibility rigor. Steps included (1) resource collection, (2) creation of eligibility criteria, (3) resource organization by topic, (4) additional content identification, (5) finalize resource selection, and (6) website testing and launch. Website visits were tracked, and a 20-item survey about website usability and utility was sent to Veterans Affairs tele-geriatrics interdisciplinary specialty care groups.

**Results:**

Following website development, the dementia caregiver resource website was launched in February 2022. Over the first 9 months, the site averaged 1100 visits per month. The 3 subcategories with the highest number of visits were “general dementia information,” “activities of daily living,” and “self-care and support.” Most (44/45, 98%) respondents agreed or strongly agreed that the website was easy to navigate, and all respondents agreed or strongly agreed that the resources were useful.

**Conclusions:**

The iterative process of creating the dementia caregiver resources website included continuous identification, categorization, and prioritization of resources, followed by clinician feedback on website usability, accessibility, and suggestions for improvement. The website received thousands of visits and positive clinician reviews in its first 9 months. Results demonstrate that an expert-vetted, nationally, and remotely available resource website allows for easy access to dementia education for clinicians to provide for their patients and caregivers. This process of website development can serve as a model for other clinical subspecialty groups seeking to create a comprehensive educational resource for populations who lack easy access to specialty care.

## Introduction

The proportion of the world population that is aged over 60 years old is projected to almost double between 2015 and 2050 [1], and with it, the prevalence of dementia and the number of families caring for people with dementia will also increase. About 11 million Americans (70% female) currently provide 18 billion hours of unpaid dementia care per year [2], many without formal dementia education or support. These informal dementia caregivers, such as friends and family members, generally do not have the knowledge or expertise to handle the myriad challenges dementia caregiving can present. This contributes to the high rates of caregiver stress and burnout, often leading to institutionalization for the person with dementia [3-5]. While clinicians (broadly defined to include all health care professionals who care for patients [6]) recognize that supporting the family caregivers of their patients with dementia is important, most do not have the time or ready access to the best educational resources for management of the varied aspects of dementia care (eg, activities of daily living, instrumental activities of daily living, safety, and navigating neuropsychiatric symptoms) at all stages of the disease. Therefore, clinicians require a compendium of high-quality, expert-reviewed resources that are easily available to them to supplement their care and to address common caregiver questions throughout the disease progression.

While some health care systems have dedicated dementia specialists or robust community dementia support, the majority do not [7]. Moreover, people with dementia and their caregivers living in rural areas or experiencing low socioeconomic status face additional barriers to accessing specialty dementia care, education, and support [8]. This often leaves caregivers to find dementia education on their own or rely on recommendations from their primary care team. Many caregivers turn to the internet to find information about caring for someone with dementia and for caregiver support. However, web-based resources are not necessarily scrutinized to ensure information accuracy or to consider the health literacy skills, technology skills, and cognitive and sensory skills of the target audience [9,10]. Similarly, because information is placed in varied locations with varying degrees of quality, cost, and access, this search can be confusing for many caregivers, leading to an increase in misinformation and further caregiver strain [11-14]. Currently, there are hundreds of websites offering advice regarding dementia; however, this plethora of choices can often be confusing or inconvenient for busy clinicians or family caregivers to navigate. It is important for clinicians to be able to direct caregivers of people with dementia to high-quality, easy-to-navigate educational content housed in a single central location that has an expert clinician–curated selection of diverse resources.

Our caregiver resources website aimed to provide clinician-vetted, easy-to-access, and dementia-specific education resources that prioritized the caregiver audience and provided a comprehensive overview of the neurodegenerative disease process, with different resources for different stages of dementia. The website development team (WDT) was comprised of 6 Veterans Affairs (VA) telehealth-based clinical dementia experts based at various Geriatric Research Education and Clinical Centers (GRECCs) centers of excellence focused on serving aging veterans. Members of the WDT had combined decades of dementia experience and represented multidisciplinary backgrounds (neurology, geriatrics, nursing, social work, and occupational therapy). The WDT used an iterative strategy to gather input from interdisciplinary geriatrics clinicians with decades of experience caring for individuals with dementia and their caregivers and create a compendium of clinician-approved, web-based dementia care resources.

## Methods

### Website Creation Overview

**Figure 1:**
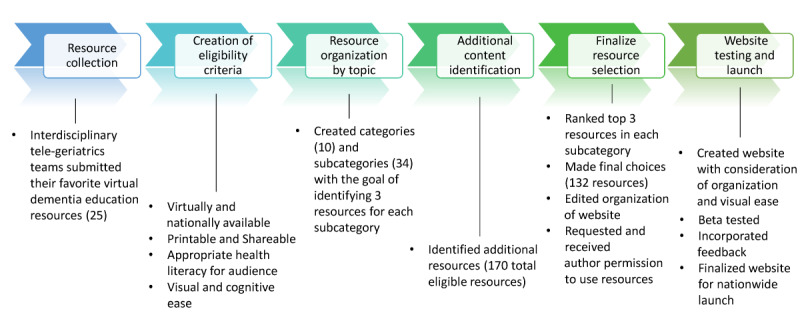
The development of the dementia caregiver resources website. A summary of the sequential steps taken over 1.5 years to develop the expert clinician-vetted dementia caregiver resources website.

The process of creating this website resource, outlined in Figure 1, included 6 iterative steps over the 1.5 years of website development to ensure resource information quality and appropriateness for informal dementia caregivers. Steps included (1) resource collection, (2) creation of eligibility criteria, (3) resource organization by topic, (4) additional content identification, (5) finalize resource selection, and (6) website testing and launch. Figure 1 shows the development of the dementia caregiver resources website and the summary of the sequential steps taken to develop the educational resource website.

#### Step 1: Resource Collection

The WDT leveraged their existing geriatrics contacts among 18 VA tele-geriatrics programs across the country and asked them to share the dementia caregiver educational resources they found most useful in their clinical practice. The only requirements for these resources were that they needed to be directed to a patient or caregiver audience and freely available on the internet remotely. This request yielded a total of 25 different web-based resources. The WDT reviewed the resources, which varied widely in content, addressing not only physical and cognitive changes related to dementia but also psychosocial, emotional, and familial or community impacts throughout the disease process. Additionally, the resources varied in overall quality, availability (some resources were directly available on the web, others required payment or a subscription, some were applicable only in a particular state or region, etc), visual clarity (challenging to read, difficult to print out, etc), length, format, and consideration for audience. The WDT used these 25 resources as their starting point for Step 2.

#### Step 2: Creation of Resource Eligibility Criteria

Based on a thorough review of resources collected in Step 1, the WDT developed specific eligibility criteria for selecting additional resources for the website. Eligibility criteria for potential resources included the following:

1. Remotely and nationally available (not programming specific to one location or region)
2. Easily accessible in printable or sharable format (PDF, Word [Microsoft Corporation] document, video, etc)
3. Appropriate health literacy level for the family caregiver audience
4. Visual and cognitive ease (eg, large and easy-to-read font, good use of white space and images, and a lack of excessive color or low contrast)
5. Open access resources (eg, not commercially sponsored, behind a paywall, or requiring email registration)

International resources were excluded if they provided country-specific data, policy, or programming. Stand-alone resources (eg, PDFs, Word documents, and videos) were given preference over general dementia or caregiving websites, as some websites can be difficult to navigate, share, and print. Preference was given to resources that were not likely to undergo excessive changes or that seemed to require infrequent updating. While this compendium was created by VA clinicians within the United States, the WTD identified resources that were applicable to dementia caregivers, regardless of veteran status.

#### Step 3: Resource Organization by Topic

In parallel to the gathering of resources from the large national VA tele-geriatrics network and the creation of the eligibility criteria, WDT members met bimonthly and also regularly shared candidate resources with other team members through email. Similar resources were grouped into categories and subcategories, identifying important gaps in content. Categories and subcategories were formed with the goal of providing as much education on common dementia-specific challenges as possible while also trying to provide at least 3 resources in varied formats for each section. Using a category and subcategory organization allows clinicians to easily provide the right resource at the right time for a given caregiver’s dementia care needs, as well as easily display the plethora of resource topics that could impact caregivers throughout the disease process, thus offering a potential “roadmap” for caregivers. So, depending on the caregiver’s needs and interests, the clinician can tailor the website experience. Again, this process of categorization was iterative, and as the identification of resources continued, categories and subcategories were edited, added, or deleted to best organize the content. The 10 finalized categories are included in Textbox 1.

**The 10 category headers on the expert clinician–vetted dementia caregiver resources website.**

**Finalized categories**
  - Dementia overview
  - Daily activities
  - Behavior changes
  - Safety
  - Caregivers’ self-care and support
  - Brain health
  - Telehealth and technology
  - COVID-19 and dementia care
  - Comprehensive dementia care guides
  - Additional websites

Each category had associated subcategories. For example, the category of “behavioral changes” was further divided into subcategories including wandering, sundowning, anxiety, aggression, and repetitive questions. There were 34 finalized subcategories in total. While most categories had a similar organizational scheme, the categories of “comprehensive dementia care guides” and “additional websites” differed. The “comprehensive dementia care guides” category housed resources that spanned multiple topic areas and dementia stages and required more time to navigate due to the amount of information provided. These guides were given their own organizational scheme and were available for clinicians to give to caregivers or family members interested in a more in-depth resource. The “additional website” category housed general dementia websites that were deemed useful by the WDT but were made up of multiple pages requiring further link selection and thus did not meet the original eligibility criteria for simplicity.

#### Step 4: Additional Content Identification

Upon review of the initial 25 resources suggested and the WDT discussions around important categories and subcategories, gaps in various topic areas were identified (eg, brain health, dementia and nutrition, and emergency preparedness). The WDT members then embarked on a broad internet search to find additional resources that fit the eligibility criteria and filled notable gaps in the established categories and subcategories. This included an expedited review of topic areas and dementia education products from esteemed organizations, such as the Alzheimer’s Association, the National Institute on Aging, etc, which helped to organize the thorough list of categories and subcategories. The WDT continued to meet regularly to identify, vet, and organize candidate resources using the eligibility criteria and, if appropriate, add them to the compendium. At the end of the content identification process, there were 170 resources that were candidates for inclusion.

#### Step 5: Finalize Resource Selection

All 170 candidate resources were organized into categories and subcategories, with each subcategory housing between 3 and 10 vetted resources. The WDT then individually reviewed all the resources in each subcategory, and each team member ranked their first, second, and third choices based on the eligibility criteria outlined above as well as the overall quality of the resource, suitability to the corresponding category and subcategory, and perceived clinical utility for clinicians and caregivers. WDT members also identified specifically why they liked or did not like each of the resources and if they had any “honorable mentions” among those that were not ranked as their top 3 choices in each subcategory. After all resources were ranked, the team collated the rankings and chose the resources with the most first, second, and third rankings. When there were more than 3 quality options available for a given subcategory, the WDT discussed them as a group and used their clinical judgment to make the final selections. This detailed analysis of resources helped the team decide whether to add or exclude additional resources, which resulted in some subcategories having more than 3 resources. This process resulted in 3-6 quality resources per subtopic area with a variety of media formats (PDF, video, etc). There were 132 resources selected for final inclusion on the website.

Once the list of resources was finalized, the WDT reached out to the authors or organization for each resource to gain permission to display the resource link on the compendium website. The caregiver resources website only provided links to the resource, ensuring all credit for the resource went to the authors or organization. This also ensured that, if there were updates to the resource in the future, the WDT would only be responsible for providing the updated link. All resources received author permission.

#### Step 6: Website Testing, Launch, and Evaluation

The WDT, in collaboration with the VA tele-geriatrics website developer, created the structure, organization, and overall look of the website. The initial target end users of this website are clinicians caring for people with dementia, although some clinicians may share the whole website (as opposed to specific resources) with caregivers. Therefore, additional care was taken when choosing resources to consider caregivers of people with dementia (who are often older adults with age-related sensory challenges). This included being mindful of category and subcategory organization, language used, font size, white space, photo content, thumbnail images to ensure inclusivity and demonstrate diversity, etc.

A link to the beta version of the website was sent nationally through email to 60 geographically diverse VA tele-geriatrics specialists who attended a meeting in which the website goals were described. The email also solicited feedback on content, visual appeal, usability, and organization. All feedback was discussed by the WDT and led to further modifications to the website’s appearance and organization. The finalized website launched in January 2022 through an email announcement to the larger VA tele-geriatrics interdisciplinary specialty care group (119 members) who were asked to share the website with their colleagues inside and outside the VA network. Additionally, to increase visibility, the WDT also described the website creation process and demonstrated the use of the website in a case-based national webinar to an audience of 117 clinicians.

A 20-item survey containing a combination of quantitative and qualitative questions was established to gather interdisciplinary clinician feedback on the website’s usability and accessibility and to solicit suggestions for improvement. Survey questions and design were informed by a review of literature on evaluation of health care resources and education websites and content [15-20]. The survey was implemented on the Survey Monkey (Momentive Global Inc) platform and disseminated in the same email as the website launch announcement as well as during the national webinar. A request to complete the survey regarding the resource website was sent through email to all members of the VA tele-geriatrics interdisciplinary specialty care group as well as the 117 attendees of the case-based national webinar. Email recipients were also invited to forward the email with the website link and survey to any colleagues they deemed appropriate. Because of this, it is unknown how many people received the survey link. The survey was open from September 14, 2022, to November 1, 2022.

### Ethical Considerations

This work was conducted as part of the VA Office of Rural Health-funded “GRECC-Connect” program education and evaluation core activities and was determined to be quality improvement by the VA Bedford Healthcare System Institutional Review Board, not human subjects research. Participants were invited to take part in the optional survey through email. Participation was voluntary and no compensation was provided. Individual participation was not tracked, and individually identifiable data were not collected.

## Results

### Final Website

**Figure 2:**
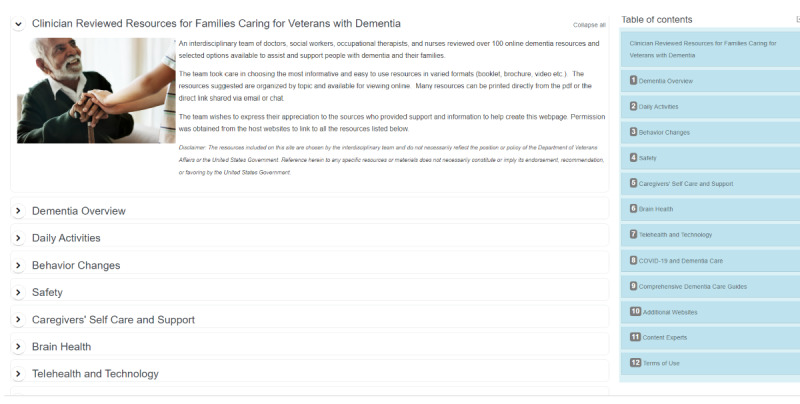
A screenshot of the landing page for the dementia caregiver resources website, a remote compendium of expert-vetted, web-based dementia educational resources for clinicians to provide to caregivers of individuals with dementia.

Figure 2 shows a section of the landing page for the dementia caregiver resources website and highlights the table of contents and organizational scheme. The website is housed under the VA Geriatric Scholars website [21].

The website includes 10 categories and 34 subcategories. A table of the final website organization, as well as the number of resources available and the number of visits by subcategory in the first 9 months after launch, is outlined in Table 1.

**Table 1:** Organizational structure and website visit count for categories and subcategories within the expert clinician-vetted dementia caregiver resources website. The caregiver resources website chart shows the finalized categories and subcategories, the number of resources in each subcategory, and the total number of visits for each subcategory over a 9-month period.

Categories			Number of resources available, n		Total number of visits over 9 months, n
**Dementia overview**					
	General dementia information	6		625	
	Non-Alzheimer dementia	3		326	
	Dementia research	3		235	
**Daily activities**					
	Activities of daily living	4		450	
	Medication	3		282	
	Activity participation	3		303	
	Traveling	3		252	
	Driving	3		306	
	Sex and intimacy	3		353	
	Sleep	5		250	
	Nutrition^a^	5		175	
**Behavior changes**					
	Anxiety and depression	4		323	
	Delirium	2		250	
	Sundowning	4		315	
	Wandering	3		274	
	Aggression and agitation	4		336	
	Hallucinations and paranoia	4		285	
	Repetitive and inappropriate behaviors	5		251	
**Safety**					
	Home safety	5		313	
	Firearm safety	2		227	
	Falls prevention	3		267	
	Emergency preparedness	3		233	
**Caregivers’ self-care and support**					
	Self-Care and support	4		492	
	Resources and programming	4		274	
	Legal, financial, and employment support	5		271	
	Goals of care or end-of-life	3		232	
	Communication	4		289	
	Long-term placement	4		217	
Brain health			4		386
Telehealth and technology			6		256
COVID-19 and dementia			3		219
Comprehensive care guides			2		365
**Additional websites**					
	Websites	11		266	
	Video series	2		244	

^a^The nutrition category was added after the website went live; therefore, only 6 months are accounted for in the reported number of visits over a 9-month period.

### Website Usage

In its first 9 months after launch, the website averaged 1100 visits per month between February 2022 and October 2022. A data assessment was completed to determine the number of visits each subcategory had over 9 months (Table 1). Website users frequented the subcategories of general dementia information (625 visits), activities of daily living (450 visits), and self-care and support (492 visits) the most often throughout the 9-month assessment period. This information is supported by the evaluation survey, where participants were specifically asked what resources were most useful to them. Many participants ranked the larger categories of dementia overview, daily activities, and caregivers' self-care and support in their top 3 most used resource categories.

### Survey Results

A total of 60 survey responses were recorded, with each question reported receiving a response from 72% (43/60) or more of the respondents. Survey responses came primarily from clinicians in the fields of nursing, pharmacy, medicine, social work, and rehabilitation. The number of years of clinical practice for respondents ranged from 0-5 years to 30+ years, with 65% (39/49) reporting 10 years or more in the field. A total of 75% (37/49) of respondents reported their caseload was comprised of 50% or more patients aged older than 65 years.

All respondents reported that it was likely or very likely that they would recommend the website to a colleague in the future, and almost all respondents (51/52, 98%) stated that it was likely or very likely that they would recommend the website to a patient or caregiver. To further assess the reach of the website, participants were asked who they had shared the website with. A total of 51% (25/41) of respondents reported that they primarily shared it with family caregivers, 67% (33/49) with colleagues at the VA, and 16% (8/49) with colleagues outside of the VA. A total of 18% (9/49) of respondents stated that they shared directly with their patients and 14% (7/49) with their personal friends and family. Respondents indicated they had shared the resource website by all the following mechanisms: sharing the overall web page link electronically, sharing links to specific resources electronically, downloading and saving specific resources to send electronically in the future, printing out resources and either handing or mailing them to their recipients, and adding links to resources in their care plan.

A total of 98% (44/45) of survey respondents either agreed or strongly agreed that the website was easy to navigate. Similarly, all respondents either agreed or strongly agreed that the resources on the website were useful for their clinical practice. When asked if the website was easy to find, 77% (34/44) agreed or strongly agreed that the website was easy to find. Survey questions allowing for free-text responses yielded further insight regarding clinician’s overall impressions of the utility of the website and allowed for suggestions for website improvement.

When asked for suggestions on how to improve the website, many respondents identified additional resource subcategories that they thought would be useful, such as resources on death and dying, exercise, and speech pathology (specifically cognition, communication, and swallowing). They also requested more resources offered in video format.

While the website received the majority of positive feedback, the limitation most identified by survey respondents was the inability to easily find the website. Participants stated that they had trouble finding the website through search engines such as Google and wished it were more accessible or visible to those that may not have the direct link (Textbox 2).

**Qualitative responses to open-text questions regarding overall impressions of the dementia caregiver resources website and suggestions for improvement.**

- **The majority of comments regarding overall impressions of the website were positive.**
  - “It is amazing and very comprehensive. Great place to start for someone that is just beginning to be a caregiver for someone with dementia!”
  - “Better than Google for finding information.”
  - “It is an excellent resource with a well-rounded variety of well vetted information from clinical experts.”
  - “It’s everything we need in one space!”
  - “Our caregivers are starving for this information.”
- **Some respondents described the ways in which they had successfully used the website in their clinical practice.**
  - “I facilitate a dementia educational class for caregivers and share website with them. It has helped them access additional information.”
  - “(I) provided link and or printout to caregivers, as part of education for caregivers in order to manage dementia related behaviors and self-care.”
- **Some participants described the ways in which they had trouble finding the website.**
  - “I couldn’t find it by Googling. I only found it by using the link provided.”
  - “I wish it showed up just from a Google search so I could find it more easily.”

## Discussion

### Overview

This paper describes the iterative process of creating the dementia caregiver resources website, a compendium of geriatrics clinician-approved resources presented in an organized, user-friendly format, as well as feedback regarding the website’s usability, accessibility, and suggestions for improvement. The 6-step development process can serve as a model for other clinical subspecialty groups seeking to create comprehensive educational resources. The success of the dementia caregiver resources website is evidenced by the number of visits to the website and the positive clinician reviews received within the first 9 months of its launch.

Previous research has found that family caregivers are using a variety of web-based resources to look for education on the diagnosis and progression of dementia, including searching the internet, reading informal journals or news articles, social media, watching TV, etc [22]. However, even with caregivers receiving information from varied sources, most caregivers expect their health care team to provide them with educational recommendations or reliable resources as a starting point. Many caregivers express confidence in their health care team but report that it can be challenging to connect with them due to clinician’s or caregivers’ busy schedules [23-25]. Furthermore, due to the degenerative nature of dementia, which causes a continuous decline in function, caregivers regularly require education on a variety of developing symptoms [26-28]. This points to a gap the caregiver resources website fills in providing vetted resources, all in one space, for clinicians to give to caregivers whenever needed without requiring additional health care visits.

Similarly, ensuring the topic areas and resources chosen would meet the needs of caregivers and families throughout the dementia spectrum was an important criterion during the process of creating and evaluating this web-based resource. The WDT’s process of continuous identification and prioritization of quality resources, along with iterative assessment of knowledge gaps and reorganization of topics and subtopics, allowed for a breadth of content areas that matched caregivers’ information-seeking trends. These trends show caregivers seek information on disease-specific diagnosis and treatment; disease-focused health care services; caregiver support and self-care; general caregiving skills; and, to a lesser degree, financial and legal information and accommodations [28-30]. Similarly, once the website launched, the team received additional confirmation from the evaluation that the content and organizational structure provided on the website were of great use to caregivers and health care teams alike.

Given the lack of geriatrics specialty care and trained geriatricians available, with even less access to specialty care for rural caregivers, the dementia caregiver resources website can provide resources and education to bridge the gap between specialty medical appointments or for those who are unable to see a geriatrics clinician due to location, limited availability, etc. Finally, those involved in dementia care can use the website to easily find appropriate information at any stage of the disease process to address the specific concerns the caregiver is facing at any given time—in other words, the right information at the right time.

### Limitations

There were limitations in the creation and dissemination of the dementia caregiver resources website. The goal of this clinical project was to provide quality resources as quickly as possible to clinicians to then give to their informal dementia caregiving audience. Some clinicians reported sharing specific resources with caregivers, while others reported sharing the website in its entirety. While caregiver feedback was not initially gathered (due to the intended end users being clinicians and the general scope of the project), caregivers were key recipients of the educational information, and therefore, not gathering their input could be a potential limitation of the project. The next steps of this project will include an assessment of caregiver perspectives regarding the website. Additionally, the content does not include all available dementia resources, as it is limited to those that the WDT could review and select within the available time frame of the project.

Another limitation of website dissemination was the location of the website. The website was not easily searchable through common search engines such as Google or Bing, and because it was housed within the GRECC-Connect parent website, it was even more difficult to find. Therefore, to disseminate the website, the end user had to be given the direct URL or know where to look within the GRECC-Connect parent website. Additionally, those in highly rural areas may have difficulty viewing certain resources, such as videos, on the website due to decreased access to high-speed internet. However, the WDT made an effort to ensure that each subcategory had options that did not require high-speed internet access, such as PDFs and Word documents. These challenges clearly limit the number of caregivers who will be able to easily access the website’s information, which further decreases the ability to accurately assess the impact of the website in future evaluations. Lastly, because we provide links to resources that reside on other content creators’ websites, there is the possibility of a change in access or content that is out of our control. The WDT has supplied their contact information so that end users can alert the team to difficulties with accessing the resources and so that visitors can suggest additional resources.

### Conclusion

This article describes the iterative process of creating and evaluating the dementia caregiver resources website, a compendium of geriatric expert-approved resources for clinicians to provide targeted education to family caregivers of people with dementia. While there is a plethora of web-based dementia caregiving information available, there lacked a single web-based compendium that housed a plethora of quality resources in an organized fashion, so that navigation was not confusing or inconvenient for busy clinicians. The WDT identified and organized the highest quality resources available that provided education on a wide variety of topics impacting people with dementia and their caregivers. Given the growing number of people with dementia and the heavy reliance on family caregivers without access to specialty dementia care, clinicians benefited from this single-site compendium. The dementia caregiver resource website received thousands of visits and positive clinician feedback, including 98% (44/45) of survey participants agreeing that the website was easy to navigate and all respondents agreeing that the resources were useful, demonstrating it to be a valuable resource for clinical care. Moving forward, there is potential for caregivers to navigate through the whole website without clinician direction to specific resources. Therefore, the next steps include gathering caregiver feedback and perspectives on the website. Clinicians can refer to the free, publicly available dementia caregiver resources website for expert-reviewed, organized, and quality resources [21]. This process can serve as a model for clinical subspecialty groups, creating comprehensive educational resources available for other populations who lack easy access to specialty care. For those developing a web-based resource that is accessible to both clinicians and older adults themselves, it may be beneficial to include experts in universal website design, specifically for older adult end users, from the beginning of the process to ensure a quality user experience for the broadest audience.

## Appendix

Multimedia Appendix 1 Dementia Caregiver Resources website survey. Survey questions and answer choices, percentage of respondents, and number of respondents reported. Survey questions did not require an answer to submit the survey therefore creating variation in the sample size for each question.

## Abbreviations

GRECC: Geriatric Research Education and Clinical Center
VA: Veterans Affairs
WDT: website development team

## Data Availability

All data generated or analyzed during this study are included in this published article (and its appendices).
