# Supplementary material for: Development and Evaluation of a Clinician-Vetted Dementia Caregiver Resources Website: Mixed Methods Approach
Source: JMIR Form Res. 2024 Apr 4;8:e54168. doi: 10.2196/54168 (PMC11027049; doi:10.2196/54168)
Supplement: Multimedia Appendix 1 [file formative_v8i1e54168_app1.pdf]

	Survey Questions	Percentage of Respondents			Number of Respondents		
**Q1**	**How did you first hear about the website?**			**100%**			**60**
	a. Coleagues at the VA			61%			37
	b. Colleagues outside the VA			0%			0
	c. Your organization at large (via email lists/listservs, TEAMs channels, educational webinars etc.)			32%			19
	d.Patients/caregivers			0%			0
	e. Family/friends			2%			1
	f. Social media connections			0%			0
	g. Other			5%			3
**Q2.**	**How often do you use the Dementia Caregiver Resources website?**			**100%**			**60**
	a. Every day			0			0
	b. Often (1x per week)			17%			10
	c. Sometimes (1-2x per month)			42%			25
	d. Rarely (1x every few months)			32%			19
	e. Never			10%			6
**Q3**	**The website is easy to navigate.**			**75%**			**45**
	a. Strongly agree			42%			19
	b. Agree			56%			25
	c. Disagree			2%			1
	d. Strongly agree			0%			0
**Q4**	**the website is easy to find.**			**73%**			**44**
	a. Strongly agree			18%			8
	b. Agree			59%			26
	c. Disagree			20%			9
	d. Strongly agree			2%			1
**Q5**	**Overall the resources on the website are useful.**			**73%**			**44**
	a. Strongly agree			55%			28
	b. Agree			36%			16
	c. Disagree			0%			0
	d. Strongly agree			0%			0
**Q6**	**Which Resources/Topics do you use/reference most often? (please select your top 3)**			**75%**			**45**
		**1st**	**2nd**	**3rd**	**1st**	**2nd**	**3rd**
	a. Dementia Overview	22%	0%	13%	10	0	6
	b. Daily Activities	9%	16%	18%	4	7	8
	c. Behavior Changes	20%	31%	33%	9	14	15
	d. Safety	16%	11%	13%	7	5	6
	e. Caregivers' Self-Care and Support	24%	16%	13%	11	7	6
	f. Brain Health	2%	2%	7%	1	1	3
	g. Telehealth and Technology	0%	4%	2%	0	2	1
	h. COVID-19 and Dementia Care	0%	2%	2%	0	1	1
	i. Comprehensive Dementia Care Guides	7%	16%	13%	3	7	6
	j. Additional Websites	0%	0%	2%	0	0	1
**Q7**	**Are there resources you would add to the website?**			**72%**			**43**
	a. yes			12%			5
	b. no			88%			38
**Q8**	**How do you use the website. (select all that apply)**			**75%**			**45**
	a. Personal education			62%			28
	b. Education for your colleagues or others in your organization			49%			22
	c. Educaiton for your patients/caregivers			76%			34
	d. As a part of a clinician care plan			31%			14
	e. Other			0%			1
**Q9**	**How often have you shared this website?**			**85%**			**51**
	a. Every day			2%			1
	b. Often (1x per week)			14%			7
	c. Sometimes (1-2x per month)			27%			14
	d. Rarely (1x every few months)			33%			17
	e. Never			24%			12
**Q10**	**Who have you shared this website with? (select all that apply)**			**82%**			**49**

	a.Patients	18%	9
	b. Caregivers	51%	25
	c. Colleagues at the VA	67%	33
	d. Colleagues outside the VA	16%	8
	e. Your organization at large (via email lists/listservs, TEAMs channels, educational webinars etc.)	14%	7
	f. Family/friends	14%	7
	g. Social media connections	0%	0
	h. Other	6%	3
**Q11**	**How have you shared the website information? (select all that apply)**	**82%**	**49**
	a. Shared the whole webpage electronically	37%	18
	b. Shared links to specific resources electroncially	41%	20
	c. Download and saved specific resources to send electroncially	35%	17
	d. Printed out specific resources and handed or mailed to your recipient	14%	7
	e. Added to clinical care plan	27%	13
	f. Other	8%	4
**Q12**	**How likely are you to recommend this website to a colleague in the future?**	**87%**	**52**
	a. Very likely	65%	34
	b. Likely	35%	18
	c. Unlikely	0%	0
	d. Very unlikely	0%	0
**Q13**	**How likely are you to recommend this website to a patient/caregiver in the future?**	**87%**	**52**
	a. Very likely	65%	34
	b. Likely	33%	17
	c. Unlikely	2%	1
	d. Very unlikely	0%	0
**Q14**	**Please indicate your clinical background. (select one)**	**82%**	**49**
	a. Nursing	20%	10
	b. Pharmacy	4%	2
	c. Physician/NP/PA	24%	12
	d. Psychology/Neuropsychology	0%	0
	e. Social Work	16%	8
	f. Occupational Therapy	10%	5
	g. Physical Therapy	4%	2
	h. Rehab-other	6%	3
	i. Other	14%	7
**Q15**	**Please indicate the zipcode of the facility you practice out of. If multiple facilities, please indicaate the zip code of the facility you spend the most time at.**	**77%**	**46**
**Q16**	**How many years have you been practicing?**	**82%**	**49**
	a. 0-5 years	6%	3
	b. 6-10 years	29%	14
	c. 11-20 years	22%	11
	d. 21-30 years	29%	14
	e. More than 30 years	14%	7
**Q17**	**Approximately what % of your patients do you consider to be rural?**	**82%**	**49**
	a. None	4%	2
	b. Less than 25%	28%	14
	c. 25%-50%	31%	15
	d. 50%-75%	20%	10
	e. 75% +	10%	5
	f. None	8%	4
**Q18**	**Approximately what % of your patients do you see via video telehealth (CVT/VVC)?**	**82%**	**49**
	a. None	22%	11

	b. Less than 25%	24%	12
	c. 25%-50%	16%	8
	d. 50%-75%	14%	7
	e. 75% +	10%	5
	f. None	12%	6
**Q19**	**Approximately what % of your patients are >65 years old?**	**82%**	**49**
	a. None	0%	0
	b. Less than 25%	2%	1
	c. 25%-50%	10%	5
	d. 50%-75%	16%	8
	e. 75% +	60%	29
	f. None	12%	6
**Q20**	**If you have shared or used the website, please tell us an anecdote/example/story from that experience and add additional suggestions or comments**	**17%**	**10**
